# An Assessment of Chemical Diversity in Microbial Natural Products

**DOI:** 10.1021/acscentsci.5c00804

**Published:** 2025-08-12

**Authors:** Roger G. Linington

**Affiliations:** Department of Chemistry, 1763 Simon Fraser University, Burnaby, British Columbia V5A 1S6, Canada

## Abstract

Natural products continue to play important roles in biomedical, agricultural and ecological science. Yet despite ongoing advances in “omics” technologies, including genomics, transcriptomics, phenomics and metabolomics, there is still no clear consensus on the scope and scale of chemical diversity in the natural world. The evolution and maturation of chemical databases for natural products offer opportunities to explore this question from a range of different perspectives. This Outlook will use data from the Natural Products Atlas to examine rates of similarity and variation among biosynthetic classes of molecules, to explore how structure can be related to function, and to examine the scope and scale of new scaffold discovery in the current era of natural products science. It presents an examination of known chemical diversity, investigates what this diversity can tell us about potential translational applications, and explores how current knowledge informs what we might expect to discover in future studies.

## Graphical abstract

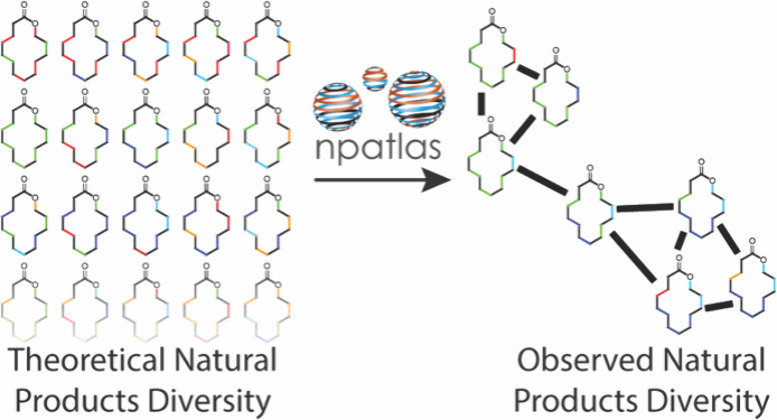

## Introduction

The natural world has long been exploited as a source of societally important small molecules. A central justification for the continued study of natural products is that nature contains a vast array of chemotypes, many of which have evolved to confer a competitive advantage to their producers. These molecules are hypothesized to be important in both ecological and translational settings, including host-microbe interactions and drug discovery.

Understanding the full landscape of chemical diversity in the natural world would influence many aspects of natural products science. For example, what is the potential return on investment of screening different types of natural product libraries (e.g., plants, sponges, fungi) against a given biological target? Which classes of natural products possess the highest potential for novel scaffold discovery? Is scaffold novelty required for identifying molecules with future value to society? If not, how does one assemble the highest diversity of scaffolds from the natural world in the most efficient format? This in turn raises interesting philosophical questions about the state of natural products research. For example, how much of naturally occurring chemical space is captured in the existing scientific literature? What is the untapped biosynthetic capacity left to discover? And how should this coverage influence future natural products investigations?

It is very difficult to characterize and classify the unknown. In the 1980s Staley and Konopka coined the phrase the “great plate count anomaly”, to describe their observation of large discrepancies between the number of microorganisms present in water samples that could be detected by direct counting compared to the number of colony forming units that would grow on Petri plates in a laboratory setting,^1^ In a similar vein, natural products science is experiencing a “great biosynthetic gene cluster anomaly” where vastly more biosynthetic gene clusters (BGCs) have been detected in genomic DNA than there are known natural products in the scientific literature.^2,3^ It is not clear whether this is because only a small fraction of available natural products have been identified to date, because current annotation technologies fail to recognize BGCs with low sequence homologies that make similar products, because the compounds from many BGCs are not produced under laboratory conditions, or because they are expressed at such low levels that they are difficult to detect and characterize.^4,5^ Natural products diversity can therefore be viewed through two different lenses. On the one hand, increasing rates of rediscovery suggest that many scaffolds have already been encountered. By contrast, examination of genomic^2,6^ and metabolomic^7^ data and heterologous expression of targeted BGCs^8–10^ continues to suggest the presence of a diverse array of novel scaffolds.

Natural products science is experiencing a “great biosynthetic gene cluster anomaly” where vastly more biosynthetic gene clusters (BGCs) have been detected in genomic DNA than there are known natural products in the scientific literature.

Although the chemical environment is constantly evolving, at any given time point the number of chemical structures on the globe is fixed. This chemical diversity is finite and can be organized into molecular classes based on structural similarity or biosynthetic origin. On an evolutionary time scale the entire history of molecular natural products science arguably forms one such “snapshot” for isolable and structurally defined metabolites. This outlook article will use The Natural Products Atlas, a database of published microbial natural products structures,^11–13^ to examine the subject of natural products diversity from several different perspectives. It will consider both organization of compound scaffolds within the data set, and the frequency of discovery of molecules with unique carbon skeletons with the aim of stimulating discussion in this interesting and complex subject area.

## How is Chemical Diversity Defined?

Although “chemical diversity” is often used in medicinal chemistry to describe variation in chemical structure, it lacks an accepted definition or method for quantification.^14^ In many cases measures of chemical diversity convert molecular representations of structures (i.e., graph representations where atoms (nodes) are connected through one or more bonds (edges)) into an array of values (a “fingerprint” or molecular encoding) where each entry describes the presence or absence of a specific structural attribute (e.g., functional groups in a list).^15^ These fingerprints can then be compared to one another, with more similar fingerprints indicating more similar structures.^16,17^ Grouping using these approaches is influenced by two factors. First, the fingerprinting method influences which functional groups are detected/prioritized. Second, the method used for scoring fingerprint similarity influences group membership.

Without clear definitions of chemical similarity and chemical diversity, assessment of chemical space becomes a subjective exercise. Taken to an extreme, it is possible to select fingerprinting methods and similarity score cutoffs that indicate that all natural products are structurally unrelated, or conversely that all natural products are part of one structural “super family”. However, chemical similarity methods can offer powerful insight into questions surrounding chemical diversity in nature if appropriately optimized. Recent examples include models for automatically assigning biosynthetic class based on structure^18^ or determining natural product “likeness”,^19^ examination of functional group distributions in natural and synthetic molecules,^20^ and several studies examining structural differences between source organism types.^16,21–23^

This article will use chemical similarity scoring to explore topics surrounding chemical redundancy and structural novelty in natural products. This approach is subject to several limitations, not least of which is that it is only possible to analyze molecules whose structures have previously been determined. Therefore, while these analyses can inform our thinking on known chemistry from nature, they cannot directly inform our understanding of future discovery potential, or the evolutionary basis of this diversity. By contrast, genomic analysis of biosynthetic potential can sometimes provide information about the evolutionary origins of BGCs but is typically not able to predict precise chemical structures and cannot determine whether BGCs are functional under a particular growth condition.

## The Similarity Landscape for Microbial Natural Products

The Natural Products Atlas uses the Morgan method (radius 2) for fingerprinting and the Dice metric (cutoff = 0.75) to score similarity between fingerprints. Applying these methods to the full database (36,454 compounds, version: v2024_09) generates 4,148 clusters containing two or more compounds which together contain 30,094 compounds (82.6%). The median cluster size is 3, and the number of clusters containing at least five members is 1,209. Somewhat surprisingly 1,093 of these clusters are at least 95% fungal or bacterial by origin. This indicates that scaffold diversity is split cleanly along taxonomic lines, with very few examples of compound classes that are made by both source types despite the use of the same primary metabolism building blocks by both kingdoms.

This grouping method typically creates clusters whose core scaffolds are closely related, meaning that molecules with similar biosynthetic origins tend to group together. As an example, cluster 70 contains 50 compounds, subdivided into three subclusters (Figure 1A). The central subcluster (box 2) contains the pyridone-containing metabolite piericidin A1 ^24^ and 19 structurally related variants. Box 1 contains glycosylated piericidin derivatives. There is high interconnectivity both within the glycosylated variants in box 1, and between glycosylated and nonglycosylated members of this family (box 2). Box 3 contains members of the γ-pyrone-containing actinopyrone,^25,26^ yoshinone^27^ and kalkipyrone^28,29^ families. Interestingly, although the carbon skeletons of the piericidins and γ-pyrones are very similar, substitution of oxygen for nitrogen in the core heterocycle motif significantly alters the chemical fingerprint, meaning that there is low interconnectivity between these two groups with only a single compound pair linking the two subclusters.

**Figure 1:**
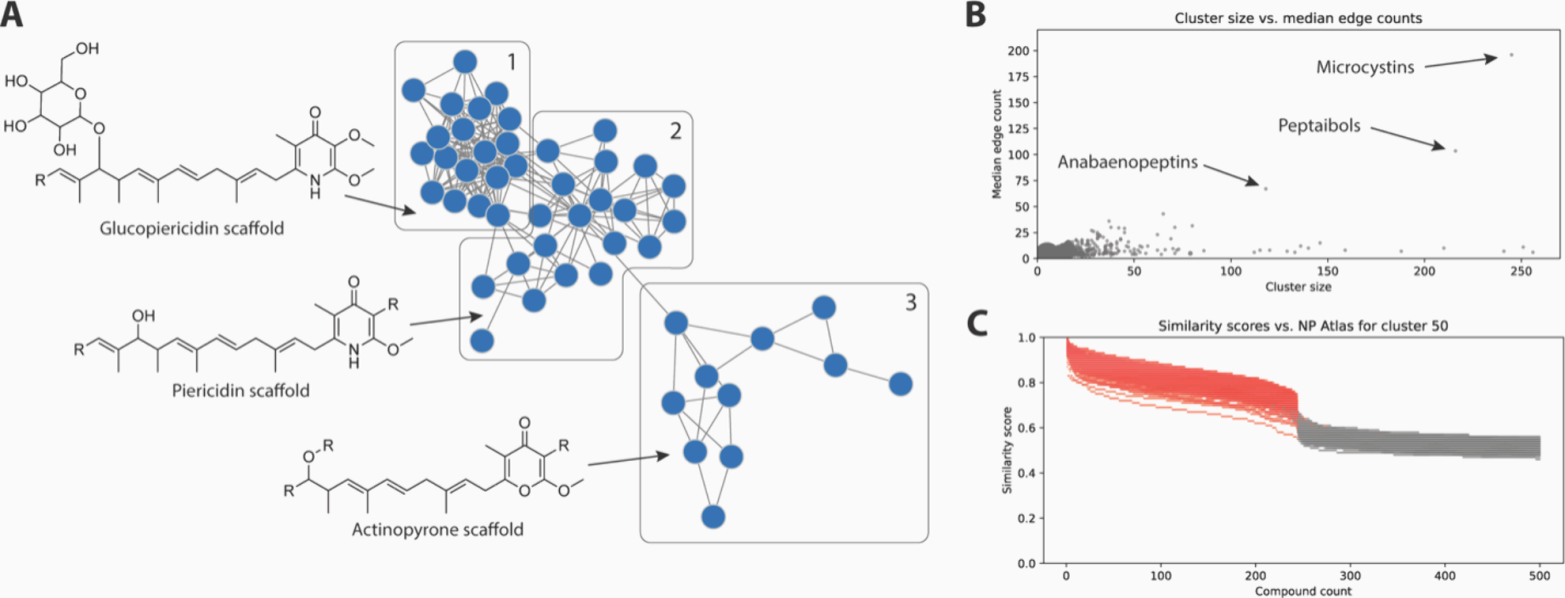
Trends in chemical diversity in the Natural Products Atlas. (A) Network diagram for cluster 70. Blue nodes are individual compounds. Edges indicate chemical similarity at or above 0.75 (Morgan fingerprint radius 2, Dice score). For a labeled version of the panel see Data Availability. (B) Plot of cluster size vs median edge count for clusters containing up to 250 members. For underlying plot data see Data Availability. (C) Plot of compound rank vs similarity score for each of the 245 members of the microcystin cluster (cluster 50) against the full NP Atlas database. Each data point represents a compound from the NP Atlas database, ranked by decreasing similarity to the test molecule (top 500 matches shown). Compounds from cluster 50 are shown in red. For underlying plot data see Data Availability.

This trait of high connectivity within clusters, but low similarity to other scaffold classes is common within the data set. One way to visualize this is to plot cluster size vs median number of connections to other compounds in the cluster (edge count). Clusters with high structural similarity (and therefore high interconnectivity) will have high median edge counts, whereas clusters containing a broader range of scaffolds will have lower median edge counts. Figure 1B presents this plot for clusters up to 250 members in size. Among these, three clusters stand out as having very high interconnectivities. These clusters contain microcystins (cluster 50), peptaibols (cluster 263) and anabaenopeptins (cluster 415), all of which are well-known compound classes in natural product science.

Microcystins are of particular societal importance due to their role as toxins in harmful algal blooms, and are subject to public health monitoring in freshwater environments.^30^ Interestingly, the median edge count (196) for this cluster is very close to the cluster size (245) indicating very high interconnectivity between compounds. This tight grouping could be because these compounds form a “island of chemical diversity” that is distinct from all other scaffolds in the data set. Alternatively, it could be an artifact of setting a similarity cutoff of 0.75 for cluster membership. To examine this question, each member of the cluster was separately compared to all compounds in the NP Atlas and the results ranked by descending similarity score. Figure 1C presents these results for all 245 members of the microcystin cluster, plotting similarity rank (*x*-axis) against similarity score (*y*-axis). Compounds are colored red if they are members of the microcystin cluster. This plot indicates that, as expected, there are very high similarity scores between members of the cluster, but that there is a steep decrease in similarity score at the boundary of the cluster, confirming that these molecules are structurally distinct from all other members of the data set.

## What Are the Drivers of Chemical Diversification in Nature?

This observation raises the question of why observed chemical diversity is sometimes localized into such structural “hotspots” rather than being more evenly distributed. Although this outlook is focused on discussing observable trends in chemical space for known molecules, it is worth briefly considering the factors that may contribute to (or define) limitations in chemical diversification. Early Darwinian proposals suggested that natural product evolution is driven by organismal fitness, and that to be retained a molecule must confer an advantage to its producer.^31–33^ Following this logic, it was argued that structures are only retained if they interact with receptors present in competitor organisms, and that molecular recognition and consequent biological function are the principal drivers of molecular diversity.^32^ Subsequently, Jones and Firn presented the “screening hypothesis” in the early 1990s^34^ which suggested that at any given time most natural products conferred no competitive advantage to the producing organism, but that by hosting a diverse array of scaffolds organisms were better prepared to respond to emerging threats to their survival. More recently, others have reframed this idea to suggest that natural products play no single defined role in host fitness, but rather provide different advantages under different scenarios, and that it is this collection of potential positive responses that drives selection (the Dynamic Matrix Chemical Evolutionary hypothesis).^35^

Separately from evolutionary drivers, chemical diversification is also influenced by the fundamental tenets of physical organic chemistry. For example, synthetic chemists have long recognized the difficulties associated with synthesizing medium-sized macrocyclic rings (8 – 11 members),^36,37^ and polyketides with these ring sizes are not commonly encountered as natural products. In a similar vein, cyclic tetrapeptides are known to be challenging to form using standard organic chemistry methods in the absence of one or more turn-inducing residues (proline, D amino acids etc.) to relieve ring strain,^38^ and are similarly rare in nature.^39^

Chemical diversification may therefore be considered to be limited by two independent factors; physical organic chemistry which influences what can be *made*, and ecological factors which influence what is *retained*. Of course, these two factors intersect when one adds the issue of biochemical pathway evolution, which places additional constraints on accessible chemical diversity. Returning to the microcystin example discussed above it is tempting to speculate that environmental function, rather than chemical inaccessibility, is the driving factor that limits scaffold diversity in this case. Within the microcystin class most amino acid residues are highly conserved, with most variation occurring at just two positions in the ring.^40,41^ Split-pool solid phase peptide synthesis and DNA-encoded libraries have demonstrated that it is possible to synthesize enormous compound libraries (∼10^6^ compounds) under standard coupling conditions,^42^ meaning that there is no intrinsic reason why diversity should be limited by synthetic accessibility. Instead, it is possible that variations at conserved positions in the ring modify the tertiary structure or other physicochemical properties, removing or reducing their environmental functions, and eliminating the selective pressure for their retention. If true, this would help to explain the large drop in structural similarity seen for molecules outside the microcystin cluster.

## Variation on a Theme is a Prevalent Trend

The analysis presented in Figure 2 suggests that, while there is a high degree of natural variation around many scaffolds, these variations are often limited to minor changes to the core pharmacophore. This observation is supported by the full cluster network (Data Availability) which contains both highly interconnected clusters, and many clusters that include highly interconnected subregions. This reinforces many of the concepts discussed in the previous section, and demonstrates that “variation on a theme” is a common phenomenon in natural products.

**Figure 2:**
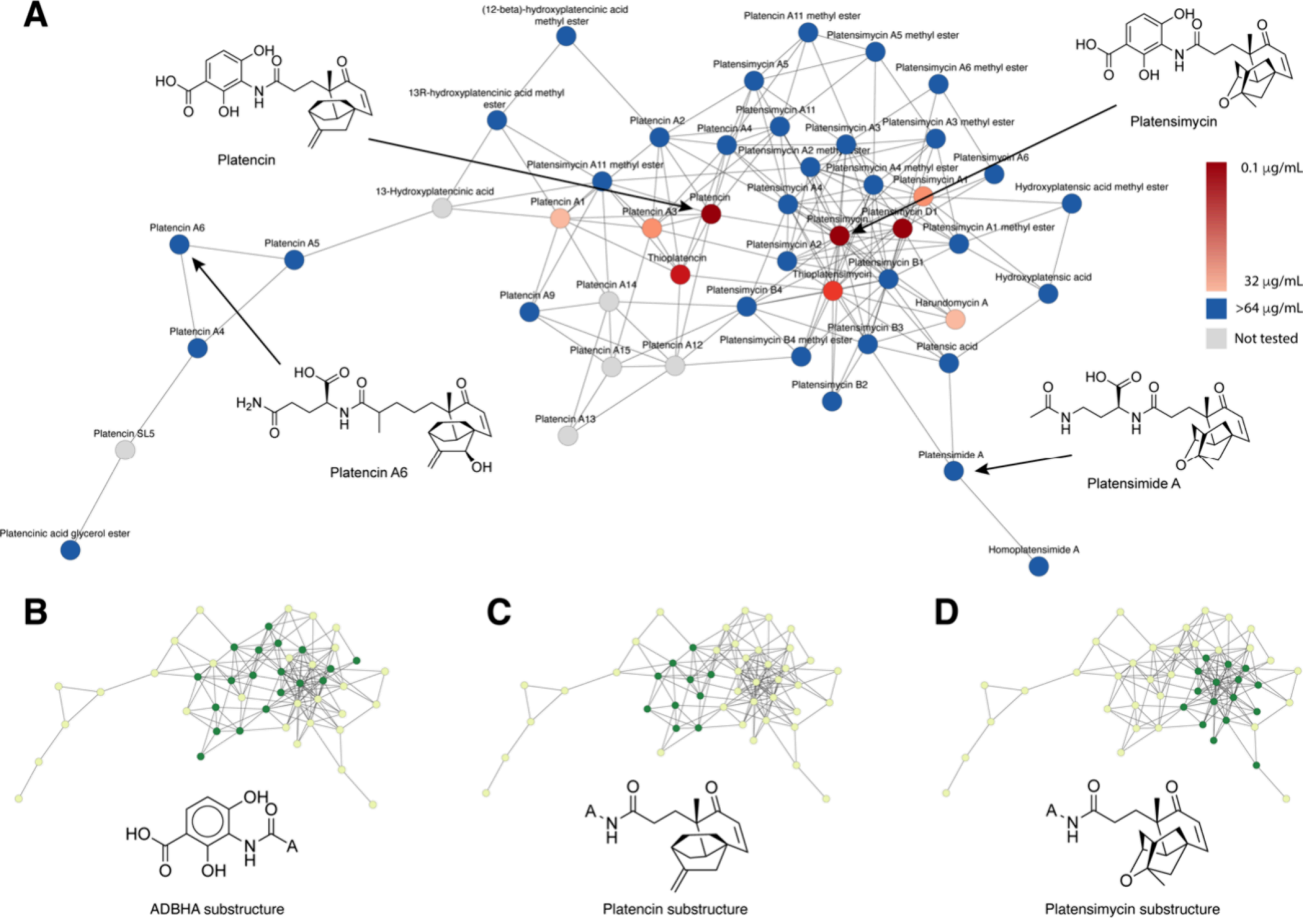
Network diagram for platensimycin/platencin cluster (cluster 112). (A) Labeled cluster network, indicating reported biological activities against the Gram-positive bacterial pathogen *Staphylococcus aureus*. (B–D) Networks indicating presence/absence of three core pharmacophore units for this cluster. Green = present, yellow = absent. MIC data derived from original isolation papers. Cytoscape file including original citations for bioactivity data included in Data Availability section.

The concept of natural pharmacophore optimization is intriguing, as it suggests that cluster interconnectivity could be used as a proxy for determining structure activity relationships for some compound classes. Considering microbially derived antibiotics, whose therapeutic function likely mirrors a key function in nature, we find that this hypothesis is supported in many cases. For example, platensimycin^43^ and platencin,^44^ two related terpene-aminobenzoic acid conjugates, are both found in the same cluster along with 46 related analogues (Figure 2A). Platensimycin and platencin both disrupt fatty acid biosynthesis through inhibition of acyl intermediates of the condensing enzymes FabB/F/H. Given their unique mode of action these compounds were the subject of intense investigation in the early 2000s. Fortunately, most derivatives were screened against the same panel of target organisms, making it possible to directly compare their biological activities. Overlaying the MIC values from broth microdilution assays against *Staphylococcus aureus* onto the cluster (Figure 2A) reveals that the analogues with the most potent antibacterial activities lie at the center of the cluster, with high interconnectivities to one another. By contrast, compounds at the edges of the cluster are uniformly inactive in this assay.

It is possible that this is because the central nodes possess the optimal structures for antibacterial activity in the environment, and that the compounds on the perimeter of the cluster are less active natural variations to the core scaffold due to shunt metabolism, post translational modification, or natural variation in biosynthetic logic. The 3-amino-2,4-dihydroxybenzoic acid (ADHBA) moiety has been shown to form key contacts in the active site of FabF.^45^ Unsurprisingly, structural variants lacking the ADHDA motif (e.g., platencin vs platencin A6 and platensimycin vs platensimide A, Figure 2A) are inactive in this assay and are not closely structurally related to many other members of the cluster. Panels 2B – D indicate the presence/absence of the ADHBA, platencin terpene and platensimycin terpene substructures. Active members of the cluster include both the ADHBA subunit and either the platencin or platensimycin terpene core, providing direct insight into the structure activity relationship for this compound class. More broadly, this observation suggests that in some cases cluster architecture may be useful in defining pharmacophores for the endogenous roles of natural products, even if those roles are unknown and no biological screening data exist.

This observation suggests that in some cases cluster architecture may be useful in defining pharmacophores for the endogenous roles of natural products, even if those roles are unknown and no biological screening data exist.

## Biosynthetic Potential vs Chemical Reality

By even the simplest approximation, the biosynthetic capacity of nature is vast. Even considering only the most common motifs present in natural product structures, the number of possible combinations is enormous. The opportunity offered by this chemical diversity is one of the principal reasons why natural products science remains an important and integral part of modern biomedical research. However, as discussed above it is difficult to estimate the true diversity of chemical space in nature.^46^ Selection pressures including the restrictions imposed by physical organic chemistry, and the requirement that molecules must convey a competitive advantage to their producing organism likely have significant influence on this diversity. This raises the question of what fraction of possible chemical space is known to exist in nature.

As an example of biosynthetic potential versus chemical reality we can consider the production of polyketide synthase-derived macrocyclic lactones. Assembly line polyketide biosynthesis involves the sequential addition of monomeric subunits to a growing polyketide chain, followed by optional modification of each subunit prior to further chain extension. The completed chain is often offloaded by intramolecular cyclization, yielding a macrocyclic lactone product. Simplifying this process to consider only the addition of two-carbon malonyl-CoA subunits, there are five possible outcomes from each chain extension step: Addition of a keto subunit, reduction of the ketone to either the R or S alcohol, dehydration of the alcohol to form an olefin, and reduction of the olefin to yield a saturated two carbon unit.

Formation of a 16-member macrocycle requires the addition of 8 monomer units, one of which is retained as a keto group to anchor the growing chain to the ketosynthase and provide the required position for macrolactonization. In principle, the remaining seven units can form 5 ^7^ (78,125) products. In reality, the number of variations is far higher because monomer addition can include a range of alternative substrates (e.g., propionate, methylmalonyl-CoA) and a wide range of additional modifications are possible at each subunit (e.g., oxidation, amination, glycosylation). It is therefore surprising that searching the Natural Products Atlas for polyketides containing a 16-member macrolactone core returns only 312 structures, grouped into 13 compound classes containing three or more members (Figure 3, A - N).

**Figure 3:**
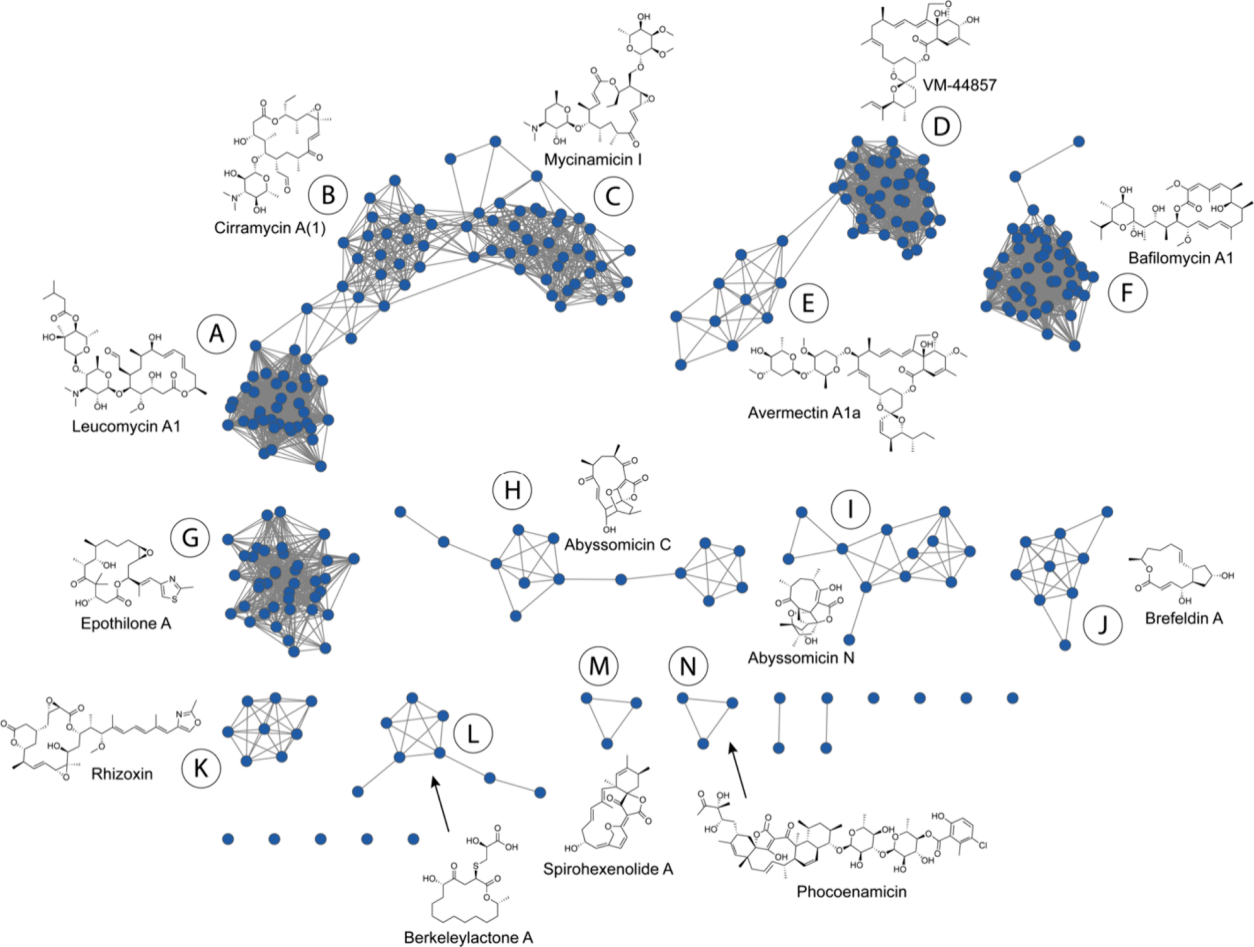
Network of all molecules from the Natural Products Atlas containing a 16-membered macrocyclic lactone. Blue nodes are individual compounds. Edges indicate chemical similarity at or above 0.75 (Morgan fingerprint radius 2, Dice score). For full labeled network see Data Availability.

Among these groups, three (A, B and C) contain very similar polyketide cores, with structural differences driven by differing sites of glycosylation around the macrolactone core. A similar situation is also observed in the avermectin class, with groups D and E differing only by the presence (E) or absence (D) of glycosylation on the ring. Lastly, two groups (H an I) are structural variations of the abyssomicin core that differ in the connectivities of intramolecular cyclizations in the central ring structure. Together these similarities reduce the number of discrete PKS classes to 10.

Closer examination of these structures indicates that many are likely constructed by polyketide synthases containing more than eight modules. Several classes contain 16 member macrolactones that are substructures of larger ring systems (VM-44857, D; avermectin A1a, E; spirohexenolide A, M; phocoenamicin, N). In addition, several structures contain long polyketide tails that extend beyond the position of lactonization (bafilomycin A1, F; rhizoxin, K). Finally, one class (abyssomicins, H and I) is constructed using the noncanonical incorporation of glycerol in place of module 8 in the assembly line. In total only four structural classes (leucomycins/cirramycins/mycinamicins, epothilones, brefeldins, berkeleylactones) derive from PKS assembly lines containing eight modules.

This limited diversity is somewhat surprising given the wide range of approaches that have been applied to natural products discovery over the past century. Natural product discovery is biased by many factors, including biological activity, chromatographic properties, chemical stability, and environmental titer among others. Nevertheless, the repeated discovery of a small subset of macrolactones by many different research teams with different research objectives over many decades suggests that these scaffolds are widely distributed in the environment and that other macrocycles are either not prioritized using typical isolation protocols, not constructed because of organic chemistry constraints, or not retained by evolutionary selection.

While 82.6% of the compounds in the Natural Products Atlas are present in clusters containing two or more compounds, the remaining 17.4% (6,360 compounds) are singletons that are structurally distinct from all other microbial natural products.

## The Long Tail of Chemical Novelty

While 82.6% of the compounds in the Natural Products Atlas are present in clusters containing two or more compounds, the remaining 17.4% (6,360 compounds) are singletons that are structurally distinct from all other microbial natural products. These include highly selective signaling molecules such as autoinducers-2 ^47^ and −3 ^48^ that play critical roles in interspecies and crosskingdom communication, as well as molecules such as geldanamycin G^49^ that are structural variants of well-known natural products (Figure 4A). This highlights one of the central advantages of natural products as a source of structural diversity, namely that unusual structures can derive either from evolution of unique chemical scaffolds (e.g., autoinducers) or from biosynthetic flexibility (e.g., geldanamycin G).

**Figure 4:**
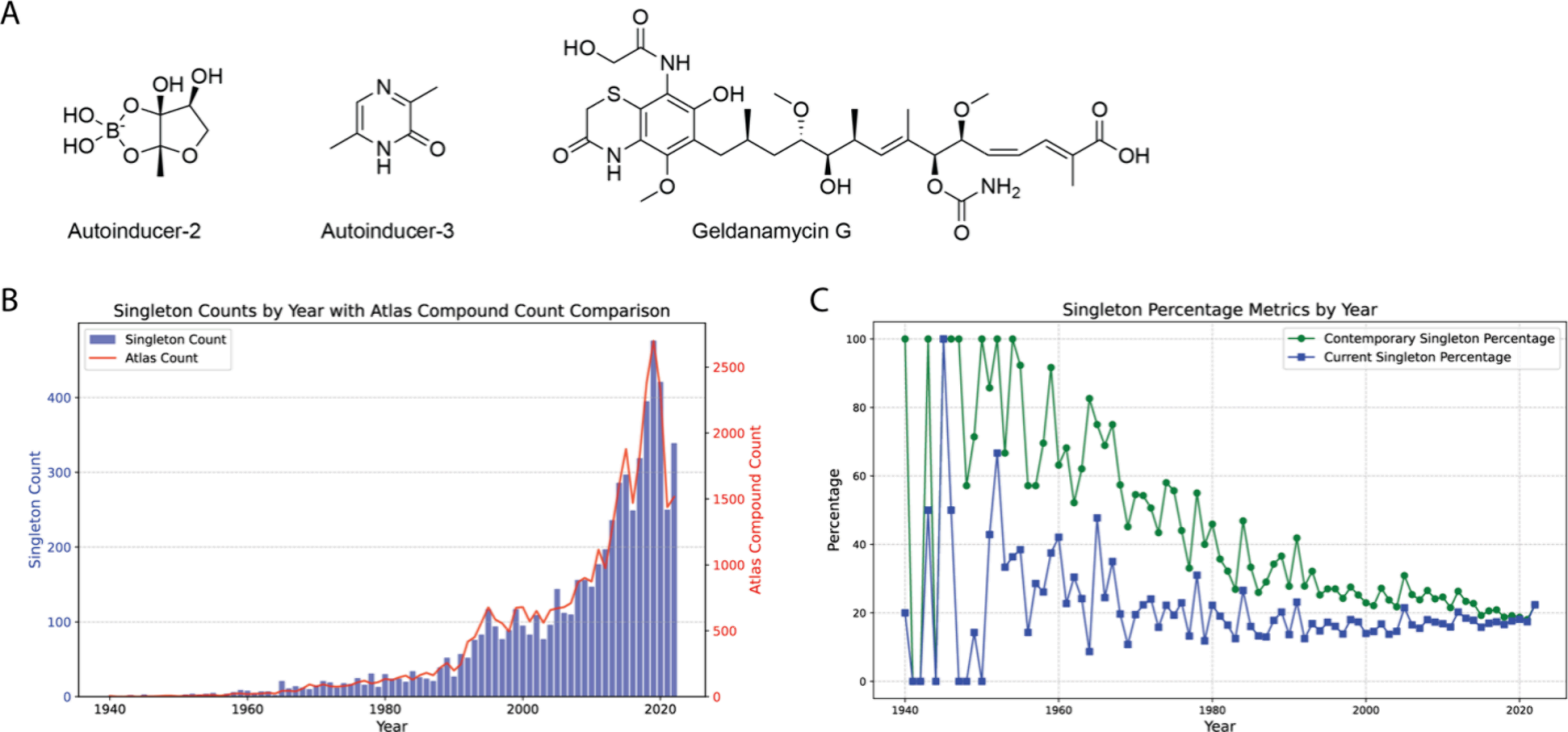
Distribution of structurally unusual natural products in the Natural Products Atlas. (A) Examples of chemical structures that form “singleton” clusters (i.e., are not related to any other structures in the database. Morgan fingerprint radius 2, Dice score ≥ 0.75). (B) Number of singleton compounds (blue) and total number of compounds (red) in the Natural Products Atlas separated by year of discovery. (C) Plot of the percentage of compounds that were singletons in the year of their discovery (green) and the percentage of compounds in each year that remain singletons when compared to all known molecules in the database (blue). For underlying data for panels B and C see Data Availability.

Arguably, this pool of structurally unique molecules is one of the most valuable and important elements of natural products research. The presence of these molecules in the data set raises several questions about their origin and distribution. For example, is structural novelty an artifact of date of discovery or are there examples of molecules that remain unique decades after their original isolation? Are most of these molecules produced by unique biochemical pathways or are they structural variations of well-established chemical classes? What is the taxonomic distribution among this group? And to what extent does niche-specific adaptation contribute to the evolution of unique/rare scaffolds?

It is well recognized that de novo structure elucidation is more challenging than determining the structures of known compound classes. This is because complex structural motifs can be difficult to identify, and nuclear magnetic resonance spectra and mass spectrometry data alone are sometimes insufficient to unequivocally determine the structures of densely functionalized molecules. By contrast, matching the spectral data of a new analogue to literature data of a related congener is significantly more straightforward, requiring the researcher only to determine the point(s) of difference with the existing structure. Following this logic, one might expect that the compounds that were unique at their time of initial discovery would become members of larger clusters as additional congeners are discovered.

To test this hypothesis, compounds were separated by year of original discovery and compared to all compounds discovered in previous years, to assess degree of novelty at the time of discovery (contemporary singleton percentage, Figure 4C, green line). Separately, compounds in each year were compared to all other compounds in the database (excluding self-comparison) to assess the current singleton percentage (blue line). As expected, rates of contemporary novelty are higher in all cases than rates of current novelty because some proportion of singletons become members of compound families in subsequent years. In the early years of the plot (1940 – 1970) the rates of contemporary novelty are often high because the total number of known natural products was low, and many discoveries were “first in class”. Over time, as the number of known natural products has grown and the annual rate of discovery has increased, the percentage of contemporary singletons has decreased, reaching a plateau of ∼20% of molecules isolated per year.

Interestingly, the percentage of singletons has remained remarkably consistent at ∼20% since the 1970s, in the face of a dramatic increase in the number of known scaffolds from microbial sources. Over the same period the percentage of molecules that were singletons in their year of discovery has gradually decreased, suggesting that many of the common classes of microbial natural products are now known. However, this analysis should not be used to suggest that the number of novel scaffolds being discovered today is decreasing. Because many compounds are isolated as families, consideration of singletons tells only part of the story. Instead, every cluster in the data set, from the very large clusters discussed in Figure 1 to the smallest clusters containing just two compounds, represent chemical space that is distinct from all other scaffolds in the data set. Both new clusters and new singletons continue to be discovered at high rates, indicating that the natural world continues to be a diverse and important source of chemical novelty. Together, the data in Figures 3 and 4 suggest that natural product structures occupy a continuum, from highly refined molecular classes with low structural variation that are widely distributed throughout the natural world, to rare scaffolds that remain structurally unique decades after their initial discoveries. This diversity analysis, coupled with the promise offered from large-scale sequencing programs suggests that there remains a wealth of new chemical matter to discover from natural sources.

## Outlook

Natural products science remains an important element of biomedical research and is central to the study of ecological systems and host-microbe interactions (e.g., human microbiome). Over the past 50 years our understanding of how these molecules are made in the environment has matured to the point that most molecules have clear biosynthetic origins. At the same time, both analytical and sequencing technologies have advanced dramatically, enabling the field to explore both the chemical composition and the biosynthetic potential of large organism libraries. What these analyses reveal is that we have an as-yet incomplete understanding of the chemistry found in nature. While many common scaffolds are now well characterized and their biosynthesis known, new class members are being discovered at high rates. For example, ribosomally synthesized and post-translationally modified peptides (RiPPs) have evolved from a biosynthetic curiosity when first discovered^50^ to a major and important class of natural products that are now found in all domains of life.^51^

Nature continues to reveal spectacular and unexpected molecules at every turn. It will be interesting to see what the next decade reveals about the chemistry of the natural world.

Beyond natural products discovery, this analysis suggests that there is significant opportunity for research programs involved in the production of “non-natural” products. The assessment of PKS diversity (Figure 3) suggests that only a small proportion of possible biosynthetic combinations are found in the environment. Ex vivo biosynthetic strategies such as transient plant expression^52^ offer exciting new opportunities for library-scale production of natural product-like libraries to generate scaffolds outside those likely to be found in nature, but which use naturally occurring biosynthetic enzymes for their production. Going one step further, there clearly remains significant value in the development of modular methods in organic synthesis to create molecules that capture the structural features of natural products without being limited by the availability and/or interoperability of biosynthetic enzymes to construct these molecules.

Another important consideration for the field is the role that informatics and information science will play in the coming decades. A previously noted, supporting the development of new technologies to better systematize the extraction of accurate structural information from genomes and metabolomes will be critical to future success.^14,53,54^ Tied to this is the importance of accurate extraction of published data into open-source databases. To be of highest value data must be curated accurately initially, and existing entries reviewed regularly for updates and corrections. Data errors can significantly reduce the accuracy of tools that use these databases for model training (e.g., predicting MS^2^ or NMR spectra) if experimental data and structures are not correctly aligned. This can further complicate analysis problems by inflating the number of candidate structures that must be considered, and including functional groups that are not actually present in nature.

As an example of the difficulties raised by this issue, in preparing this article the structure of the dichloromethyl ether containing metabolite acrodontiolamide^55^ was revisited. This structure is a singleton in the current version of the Natural Products Atlas and is the only molecule containing the unusual dichloromethyl ether functional group. Reinspection of the published ^13^C NMR data suggested a possible mis-assignment of the well-known microbial antibiotic chloramphenicol which contains a dichloromethyl ketone in place of the dichloromethyl ether. Comparison against calculated data for both candidate structures^56^ supported this reassignment, which was confirmed by comparing the published data to experimental NMR data for chloramphenicol available in the Natural Products Magnetic Resonance Database.^57^ Unfortunately, the incorrect structure can now be found in other chemical structure databases and is likely to persist for the foreseeable future, even once the entry is updated in the Natural Products Atlas, highlighting the challenge of accurate data management in this area.

Natural products science is enjoying a period of renewed prominence in both basic science and translational settings. Following over a decade of effort focused on the development of new technologies to systematize and automate information extraction from genomic, metabolomic, and screening data, natural products research is now once again the central focus of large-scale discovery programs for a range of human health and agricultural applications. This includes the formation of new companies dedicated to natural products science as well as the expansion of large-scale collaborative projects with academic/industrial natural products libraries^58–60^ Many of these efforts are including both known and novel chemical scaffolds in therapeutic development pipelines, meaning that the full range of chemical space available from nature is relevant to these programs, rather than just the much small set of novel scaffolds found each year. Lastly, expansion of investigation into new areas of taxonomic space continues to yield an exciting and unusual array of new chemotypes, many of which have no precedent in natural products science. From metagenomic sequencing and the identification of obligate symbionts,^61,62^ to the investigation of taxonomic groups that have been historically understudied (e.g., Burkholderiales^8,9^ and Acidobacter^63^), Nature continues to reveal spectacular and unexpected molecules at every turn. It will be interesting to see what the next decade reveals about the chemistry of the natural world.

## Supplementary Material



## Data Availability

Spreadsheets and Cytoscape files containing the underlying data for Figures 1–4 are available on Zenodo (10.5281/zenodo.15346913).

## Notes

Transparent Peer Review report available (PDF)
