# Supplementary material for: An Assessment of Chemical Diversity in Microbial Natural Products
Source: ACS Cent Sci. 2025 Aug 12;11(9):1536–45. doi: 10.1021/acscentsci.5c00804 (PMC12464764; doi:10.1021/acscentsci.5c00804)
Supplement: Supplementary file 1 [file oc5c00804_si_001.pdf]

oc-2025-008044.R1

Name: Peer Review Information for "An Assessment of Chemical Diversity in Microbial Natural Products"

First Round of Reviewer Comments

Reviewer: 1

Comments to the Author

This outlook by Dr. Linington describes an analysis of the chemical diversity of known natural products. There are many interesting observations presented that will help guide future natural product discovery efforts including the observation that central nodes in a network of related compounds are more likely to have higher activity, that characterized polyketides represent an extremely small fraction of the theoretical polyketide diversity, and that there is a long tail of chemical novelty.

I only have one minor concern, in the third paragraph of the introduction, Dr. Linington discusses the "great biosynthetic gene cluster anomaly" and suggests possible explanations for this anomaly as only a small fraction of natural products having been discovered or that annotation software fail to identify distantly homologous BGCs that make similar products. I agree that both of these are possible explanations for the anomaly but think that there are some other possible explanations as well:

1. Some gene clusters may be identified as BGCs but may not be functional in the sense that they are not capable of producing a product. They could be non-functional mutants of characterized BGCs, so there is some connection to the homology issue already presented by the author, but this possibility is still distinct.

2. That there are a few highly common BGC families and many rare BGC families (or even many singleton BGCs). This possibility is alluded to a little towards the end of the paragraph when discussing frequent rediscovery of known compounds and is also related to the already presented possibility that only a small fraction of natural products has been discovered, but it is not fully discussed. This possibility also seems consistent with the

observations later in that paper of the long tail of clusters with few compounds, so perhaps it is worth a bit more discussion.

Reviewer: 2

Comments to the Author

This is an excellent Outlook article that offers a timely and thought-provoking perspective on the scope and structure of chemical diversity in microbial natural products. The use of the Natural Products Atlas to analyze scaffold diversity, chemical similarity networks, and structural novelty is insightful, and the manuscript is clearly written and well organized. The figures are informative and compelling, and the text touches on many issues central to ongoing discussions in natural products research and cheminformatics.

The manuscript is absolutely suitable for ACS Central Science, and I strongly support publication. One of its key strengths is how it stimulates reflection on the frameworks we use to interpret natural product diversity. In that spirit, I would like to offer a few conceptual points—particularly from a biological perspective—that might enrich the discussion.

It seems to me that the article is written largely from a chemist's perspective, with an emphasis on structural variation and biosynthetic capacity. While this is appropriate given the topic, in my view it underrepresents the fact that natural product diversity is not solely a matter of chemical possibility—it is shaped by biological function and evolutionary selection. Microbial metabolites are not randomly assembled: they persist because they confer a selective advantage in ecological contexts (e.g., antimicrobial activity, competition, signaling).

For example, in the section Biosynthetic Potential vs. Chemical Reality, the manuscript suggests that the repeated discovery of only a few macrolactone scaffolds may reflect isolation biases or environmental distribution. While these are plausible factors, I believe the analysis overlooks a crucial point: the compounds we repeatedly observe are not just those that can be made—they are those that have been selected for due to their ecological relevance and biological activity. Evolution does not sample biosynthetic space randomly; it filters it. Acknowledging this would strengthen the interpretation of the data and make the discussion more accessible to biologists.

The same applies to the microcystin cluster example. The sharp boundary observed between this well-connected group and the broader chemical space is described in terms of fingerprinting thresholds, but could it not also reflect selective optimization? Perhaps these clusters are internally cohesive because their structures have been fine-tuned by evolution for specific functions—and other permutations were biologically less useful or actively selected against.

The discussion on singletons is also strong, and the historical analysis is illuminating. Still, I wonder whether some of these structurally unique compounds might be framed not only as biosynthetic outliers, but also as the products of niche-specific adaptation. Their rarity could reflect ecological specialization, rather than mere under-sampling or detection limits.

One sentence struck me as problematic: "…cannot determine which BGCs are functional under a particular growth condition (or ever)." From a biological perspective, the idea that some BGCs may never be functional seems unlikely. Bacteria do not generally maintain large, energetically expensive biosynthetic gene clusters if they are functionless. While many BGCs are silent under standard laboratory conditions, they are likely expressed in specific environmental contexts.

Overall, I found this article to be both rigorous and conceptually stimulating. It does an excellent job of highlighting the potential of cheminformatics tools to explore natural product diversity, I only had some minor comments.

Reviewer: 3

Comments to the Author

This manuscript on the chemical diversity of natural products written by Roger Linington is timely. The outlook provided by the author is based on the Natural Products Atlas that they lead. The idea of exploring emerging features of the chemical diversity underlying natural products research is a topic that has been neglected, with most of the work on NPs focusing on empirical trail-and-error efforts during NP drug discovery based in screening of biomolecular activities such as antibiotics. With the availability of a growing number of robust databases as the NP Atlas, and analysis workflows, the possibility of providing an outlook is much welcomed. As such, this outlook manuscripts provides an interesting analytical exercise with regards to important questions as how similar are the NPs we have

chemically (structurally) characterized to date, and what is the trend in novelty if we keep doing business as usual.

To support their opinion, the author provides analyses based on selected data-information available at the NP Atlas. This include Figure 1 about overall chemical diversity (where the interesting concept of Merdian Edge Count is introduced); Figure 2 about chemical variation amongst related classes of metabolites in relation to their cognate antibiotic activity (a well-acknowledged observation); Figure 3 on the conservation of scaffolds, yet no mention of convergent evolution; and Figure 4 about novelty discovery rates in historical perspective. I found each one of these analysis to tell an independent and interesting story. However, the interconnectivity and flow of these analysis, in my humble opinion, is lacking. convoluted and forced. Moreover, in many cases, very strong statements without support are made, often out of place and without acknowledging previous work on the nature of chemical diversity.

Below I provided some specific comments that will hopefully serve the author to better delineate the focus and narrative of this outlook paper

1. The main problem I see with the current (lack of) focus is that the aim is not clear: is this about defining trends on chemical diversity to better understand the evolution of this complex trait? Or is it about mapping chemical diversity per se to direct future efforts in NP drug discovery? These two are related but also are different questions, and fundamentally oppose in so cases, so they are not interchangeable concepts. Doing so is misleading and provides reasonable but wrong assumptions specially with regards to the first question about the evolution of chemical diversity.

2. The author themselves acknowledge that NP Atlas is biased towards what we can bioactively screen (from pharma perspective), purify and structurally characterize, yet, as mentioned in the previous point, the manuscript is plagued with evolutionary assumptions without support that most likely reflect this biased. Examples of this are the following:

a. Page 3, Lines 49-53, ...this chemical diversity if finite and can be organized into molecular classes.... On the evolutionary timescale.... forms one such snapshot. This is true for isolated and structurally resolved metabolites but not for the chemical diversity produce by living cells. See for example historical and influential opinion papers by Noel, Fischbach, etc.

b. Page 4, what defines chemical diversity evolutionary speaking is missing altogether. See Darwinian proposals by Firn and Jones, as well as Barona Gómez

c. Page 6 plus here and there, the concept of biosynthetic plasticity is not well defined, and this is because it is still not well understood. From enzyme promiscuity to epigenetics

and population level factors can all underly this, so impossible to address it with the chemically-biased NP atlas database.

d. Page 6, lines 49, ...whose therapeutic function likely mirrors the primary function in nature...., again this is a conceptual trap: the only function explored during screening captured by the NP atlas are those we humans care about. There is no such a thing as primary function as the environment is dynamic and that is what defines evolution. See the dynamic matrix chemical evolutionary hypothesis postulated by Chevrette, Barona Gómez, Hoskisson.

e. Page 8, lines 6-9, similar to previous point: the only thing that could be concluded about the relationship between BGC diversity and biomolecular activity is limited to the bioassay used, and thus cannot be extrapolated into such strong evolutionary statement(s).

f. Page 10, lines 7 – 15. Indeed, the authors are aware of the biased in place. So it is hard for me to explain why these were not more carefully examined to avoid incongruent and misleading statements about the evolution of chemical diversity.

3. Not sure where the MIC data of Figure 2 comes from and how these data was treated.

4. The author miss altogether the important role of convergent evolution during synthesis of key functional moieties, for instance the beta lactam ring. Probably this is what they refer to in Figure 2? See opinion papers by Fischbach, Walsh, Ross and others on convergent evolution.

5. Within the final Outlook section, I found the second paragraph not informative and out of place (This manuscript is not about unnatural natural products, but quite the opposite).

6. Also in this section, ref 39 is wrong. While third paragraph lacks all together any reference to previous work or ideas being discussed.

7. Page 13, last paragraph includes companies that should not be mentioned as many are omitted. Same with NP research programs. Not a good approach for an outlook paper.

Author's Response to Peer Review Comments:

oc-2025-008044 response to reviewers

I thank the reviewers for their careful and thoughtful reviews of this outlook article. The discussion was measured and detailed, and consideration of these additional viewpoints has helped me to strengthen the paper in several important ways. A central critique that ran through the reviews was that the discussion was too ‘chemistry-centric’ and did not appropriately consider evolutionary factors that influence chemical diversity. To address this criticism, I have added a new section entitled *‘What are the Drivers of Chemical Diversification in Nature?’* where I have attempted to summarize some of these key points. It is difficult to cover all aspects of this complex field in a single section, but I hope that this addition improves the balance of the article and gives readers food for thought in this area. In line with this change, I have also reviewed this aspect of the discussion throughout the text and removed or modified statements that the reviewers indicated were too strong or too narrow.

A point-by-point response to reviewer comments and questions follows.

Editor

Pull Quotes (Outlook): We encourage you to select 3 - 4 quotes from your Outlook that you would like highlighted in your paper. The quotes should be one sentence-long, unique to the Outlook and not from previously cited work. Please list your quotes at the end of the manuscript file.

Response: Addition. I have added the pull quotes as requested.

Supporting Information: Please remove the "Supporting Information" heading before your data availability statement. Supporting Information should only refer to separate files published online-only with the manuscript. Since you cited data, please change the heading to something like "Data Availability" and update any in-text references to Supporting Information.

Response: Correction. I have removed references to the Supporting Information and updated the Data Availability statement. I have also updated the files in Zenodo to the latest versions for this submission.

Synopsis: ACS Central Science requires a brief synopsis. The synopsis should be no more than 200 characters (including spaces) and should reasonably correlate with the Table of Contents (TOC) graphic. The synopsis is intended to explain the importance of the article to a broader readership across the sciences. Please place your synopsis in the manuscript file after the TOC graphic and label as “Synopsis.”

Response: Addition. I have added the synopsis as requested.

TOC Graphic: Include a TOC graphic illustrating the significance of the paper. The TOC graphic should be something that is representative of your entire work. Color schemes or illustrations typically make good choices. The TOC graphic must be original and free from any copyright issues. Confirm that all text is legible. Present the TOC graphic on the last page of the manuscript by itself. Please label the TOC as “TOC Graphic”. A caption describing the TOC is not needed. Please see more information/guidelines for TOC Graphics at the following link:
http://pubsapp.acs.org/paragonplus/submission/toc_abstract_graphics_guidelines.pdf?

Response: Addition. I have added a TOC graphic as requested.

Reviewer: 1

“This outlook by Dr. Linington describes an analysis of the chemical diversity of known natural products. There are many interesting observations presented that will help guide future natural product discovery efforts including the observation that central nodes in a network of related compounds are more likely to have higher activity, that characterized polyketides represent an extremely small fraction of the theoretical polyketide diversity, and that there is a long tail of chemical novelty.”

Response: Comment. I thank the reviewer for their positive reception of this manuscript.

“I only have one minor concern, in the third paragraph of the introduction, Dr. Linington discusses the "great biosynthetic gene cluster anomaly" and suggests possible explanations for this anomaly as only a small fraction of natural products having been discovered or that annotation software fail to identify distantly homologous BGCs that make similar products. I agree that both of these are possible explanations for the anomaly but think that there are some other possible explanations as well:

1. Some gene clusters may be identified as BGCs but may not be functional in the sense that they are not capable of producing a product. They could be non-functional mutants of characterized BGCs, so there is some connection to the homology issue already presented by the author, but this possibility is still distinct.

Response: Discussion/ Addition. This point is well taken. The question of whether or not organisms retain non-functional BGCs is still hotly contested in the field. Indeed, reviewer 2 provides the opposite viewpoint that non-functional BGCs are never retained! Rather than making a declarative statement about non-functional clusters I have added this idea to the section by stating that BGCs may not be functional under laboratory conditions, or that the products may be produced at levels too low for detection/ characterization.

2. That there are a few highly common BGC families and many rare BGC families (or even many singleton BGCs). This possibility is alluded to a little towards the end of the paragraph when discussing frequent rediscovery of known compounds and is also related to the already presented possibility that only a small fraction of natural products has been discovered, but it is not fully discussed. This possibility also seems consistent with the observations later in that paper of the long tail of clusters with few compounds, so perhaps it is worth a bit more discussion."

Response: Discussion/ Addition. I agree that this possibility should also be considered. I have restructured this section so that the discussion now begins *"It is not clear whether this is because only a small fraction of available natural products have been identified to date, because…."*. I hope that this is appropriate to satisfy this suggestion.

Reviewer: 2

"This is an excellent Outlook article that offers a timely and thought-provoking perspective on the scope and structure of chemical diversity in microbial natural products. The use of the Natural Products Atlas to analyze scaffold diversity, chemical similarity networks, and structural novelty is insightful, and the manuscript is clearly written and well organized. The figures are informative and compelling, and the text touches on many issues central to ongoing discussions in natural products research and cheminformatics.

The manuscript is absolutely suitable for ACS Central Science, and I strongly support publication. One of its key strengths is how it stimulates reflection on the frameworks we use to interpret natural product diversity. In that spirit, I would like to offer a few

conceptual points— particularly from a biological perspective—that might enrich the discussion."

Response: Comment. I thank the reviewer for their enthusiasm for this manuscript, and for subscribing to the spirit of enquiry and discussion that it was designed to foster.

"It seems to me that the article is written largely from a chemist's perspective, with an emphasis on structural variation and biosynthetic capacity. While this is appropriate given the topic, in my view it underrepresents the fact that natural product diversity is not solely a matter of chemical possibility—it is shaped by biological function and evolutionary selection. Microbial metabolites are not randomly assembled: they persist because they confer a selective advantage in ecological contexts (e.g., antimicrobial activity, competition, signaling).

For example, in the section Biosynthetic Potential vs. Chemical Reality, the manuscript suggests that the repeated discovery of only a few macrolactone scaffolds may reflect isolation biases or environmental distribution. While these are plausible factors, I believe the analysis overlooks a crucial point: the compounds we repeatedly observe are not just those that can be made—they are those that have been selected for due to their ecological relevance and biological activity. Evolution does not sample biosynthetic space randomly; it filters it. Acknowledging this would strengthen the interpretation of the data and make the discussion more accessible to biologists.

Response: Discussion/ Addition. This is an important point, and one that has been addressed in the opening paragraph above. As described, this topic is now covered by the new section on drivers of chemical diversification. I have also modified the final paragraph of this section to broaden the discussion of possible reasons for the observed narrow distribution of isolated scaffolds.

The same applies to the microcystin cluster example. The sharp boundary observed between this well-connected group and the broader chemical space is described in terms of fingerprinting thresholds, but could it not also reflect selective optimization? Perhaps these clusters are internally cohesive because their structures have been fine-tuned by evolution for specific functions—and other permutations were biologically less useful or actively selected against."

Response: Addition. I have used the microcystin case as an exemplar for how ecological considerations may also influence chemical diversity in the new section discussing ecological factors, and included additional discussion on possible drivers of selection for this compound class. I welcome feedback on this new section.

"The discussion on singletons is also strong, and the historical analysis is illuminating. Still, I wonder whether some of these structurally unique compounds might be framed not only as biosynthetic outliers, but also as the products of niche-specific adaptation. Their rarity could reflect ecological specialization, rather than mere under-sampling or detection limits."

Response: Discussion/ Addition. I appreciate this suggestion, which was overlooked in the original draft. While this section does not aim to address the question of why certain scaffolds are rare, I have modified the opening section so that the concept of specialized function is introduced to readers.

"One sentence struck me as problematic: "…cannot determine which BGCs are functional under a particular growth condition (or ever)." From a biological perspective, the idea that some BGCs may never be functional seems unlikely. Bacteria do not generally maintain large, energetically expensive biosynthetic gene clusters if they are functionless. While many BGCs are silent under standard laboratory conditions, they are likely expressed in specific environmental contexts."

Response: Correction. The counterpoint to this argument was raised by reviewer 1. As described above, rather than state a position on the issue I have moderated the text to remove the suggestion that some BGCs may be non-functional and instead added text describing how BGCs may not be functional (or products detectable) under laboratory conditions.

"Overall, I found this article to be both rigorous and conceptually stimulating. It does an excellent job of highlighting the potential of cheminformatics tools to explore natural product diversity, I only had some minor comments."

Response: Comment. Many thanks for your constructive and valuable review.

Reviewer: 3

"This manuscript on the chemical diversity of natural products written by Roger Linington is timely. The outlook provided by the author is based on the Natural Products Atlas that they lead. The idea of exploring emerging features of the chemical diversity underlying natural products research is a topic that has been neglected, with most of the work on NPs focusing on empirical trail-and-error efforts during NP drug discovery based in screening of biomolecular activities such as antibiotics. With the availability of

a growing number of robust databases as the NP Atlas, and analysis workflows, the possibility of providing an outlook is much welcomed. As such, this outlook manuscripts provides an interesting analytical exercise with regards to important questions as how similar are the NPs we have chemically (structurally) characterized to date, and what is the trend in novelty if we keep doing business as usual."

Response: Comment. The reviewer has nicely summarized the motivation for preparing this opinion piece, particularly with respect to the influence that analyzing current trends could have on future discovery efforts.

"To support their opinion, the author provides analyses based on selected data-information available at the NP Atlas. This include Figure 1 about overall chemical diversity (where the interesting concept of Merdian Edge Count is introduced); Figure 2 about chemical variation amongst related classes of metabolites in relation to their cognate antibiotic activity (a wellacknowledged observation); Figure 3 on the conservation of scaffolds, yet no mention of convergent evolution; and Figure 4 about novelty discovery rates in historical perspective. I found each one of these analysis to tell an independent and interesting story. However, the interconnectivity and flow of these analysis, in my humble opinion, is lacking. convoluted and forced. Moreover, in many cases, very strong statements without support are made, often out of place and without acknowledging previous work on the nature of chemical diversity."

Response: Comment/ Discussion. The reviewer clearly summarizes the main sections of the manuscript. It is unfortunate that the structure of the paper is not clearer to the reader. The manuscript is structured into five sections. In the first section the concept of chemical similarity is introduced, and trends in chemical similarity across the full NP Atlas dataset are discussed. In the second section the paper examines specific clusters of structures and the relationship between graph architecture and biological properties. The third section contrasts theoretical versus isolated chemical diversity, while the fourth section discusses compounds with no similarity to other members of the dataset. Finally, the fifth section offers an outlook and discussion on the topic. Therefore, the manuscript follows a discursive arc from a global view of known diversity in section 1 all the way down to consideration of individual compounds in section 5. Rather than offering a comprehensive discussion at each stage, the paper provides data-driven examples of observed trends as a mechanism to stimulate discussion on this topic.

To better inform readers about the design and structure of the paper the abstract has been updated to more clearly summarise the topics to be discussed.

“Below I provided some specific comments that will hopefully serve the author to better delineate the focus and narrative of this outlook paper

1. The main problem I see with the current (lack of) focus is that the aim is not clear: is this about defining trends on chemical diversity to better understand the evolution of this complex trait? Or is it about mapping chemical diversity per se to direct future efforts in NP drug discovery? These two are related but also are different questions, and fundamentally oppose in so cases, so they are not interchangeable concepts. Doing so is misleading and provides reasonable but wrong assumptions specially with regards to the first question about the evolution of chemical diversity.”

Response: Discussion/Addition. This point is important, as it frames many of the subsequent critiques in this review. The reviewer makes the criticism that the article does a poor job of discussing the evolutionary origins of the observed variations and limitations in chemical diversity. To clarify, the outlook is focused on published chemical structures and what this information can tell us about isolable chemical diversity from nature. It is designed to discuss ***what*** is currently known about chemical diversity from nature. It does not aim to provide answers to ***how*** this selection has been driven (evolutionary rationale), or ***whether*** the observed chemical diversity is representative of all existing natural products.

This outlook should be seen as an examination of known chemical diversity, what this diversity can tell us about potential translational applications, and how what is currently known may inform what we might expect to discover in future studies. It is hoped that this analysis will also spur further discussion in these areas and that this may in turn encourage researchers in related but separate areas to contribute similar perspectives (e.g. evolutionary drivers of diversity).

Nevertheless, as described in the opening paragraph consideration of ecological perspectives was a recurring theme among the reviewers. To address this, I have added a new section on this topic (*‘What are the Drivers of Chemical Diversification in Nature?*) and have reviewed and modified the text throughout the article to better incorporate these themes.

“2. The author themselves acknowledge that NP Atlas is biased towards what we can bioactively screen (from pharma perspective), purify and structurally characterize, yet, as mentioned in the previous point, the manuscript is plagued with evolutionary assumptions without support that most likely reflect this biased. Examples of this are the following:

a. Page 3, Lines 49-53, …this chemical diversity if finite and can be organized into molecular classes…. On the evolutionary timescale…. forms one such snapshot. This is true for isolated and structurally resolved metabolites but not for the chemical diversity produce by living cells. See for example historical and influential opinion papers by Noel, Fischbach, etc."

Response: Discussion/ Addition. Natural products research has now matured to a point where many of the central compound classes are well described. The evolutionary perspective that chemical space is boundless may be true at the molecular/cellular level, but existing data does not support the idea that continued study of the same organisms will yield an unending supply of new scaffolds in the laboratory. Indeed, the high rates of rediscovery and low numbers of novel carbon skeletons being reported suggest that isolable chemical space is becoming well described for many commonly studied organisms. This outlook paper is clear and transparent in its focus on isolated/ isolable chemistry. It is aimed at the majority of natural products practitioners for whom compound isolation and identification is a required element of any research project.

In addition to the expanded introduction section (see previous point) this paragraph has been updated to more clearly highlight this distinction.

"b. Page 4, what defines chemical diversity evolutionary speaking is missing altogether. See Darwinian proposals by Firn and Jones, as well as Barona Gómez"

Response: Discussion/ Clarification. This critique has been discussed in point 1 above. The author is indeed aware of these topics and explicitly mentions the work of Firn and Jones by name later in the manuscript. The evolutionary definition of chemical diversity is excluded in this section because this section pertains exclusively to medicinal chemistry and the characterization of diversity in chemical structures, which is of relevance to the analyses that follow, rather than commenting on what the drivers of diversity are in nature.

This distinction has been strengthened by changing the title of this section from *'What Defines Chemical Diversity?'* to *'How is Chemical Diversity Defined?'*. Additional details have also been added to the paragraph to make the focus on structural diversity as it pertains to organic chemistry clearer to readers.

"c. Page 6 plus here and there, the concept of biosynthetic plasticity is not well defined, and this is because it is still not well understood. From enzyme promiscuity to

epigenetics and population level factors can all underly this, so impossible to address it with the chemicallybiased NP atlas database."

Response: Discussion/ Correction. In this context, biosynthetic plasticity is defined as being the ability of a single biosynthetic pathway to make different but related molecules through (for example) substrate promiscuity and/or inclusion or exclusion of late-stage tailoring steps. This definition has precedent in the literature.[1–5] However, for the sake of clarity the term 'biosynthetic plasticity' has been removed throughout the manuscript and replaced with descriptions that clearly indicate structural variation from a given biosynthetic pathway.

"d. Page 6, lines 49, ….whose therapeutic function likely mirrors the primary function in nature…., again this is a conceptual trap: the only function explored during screening captured by the NP atlas are those we humans care about. There is no such a thing as primary function as the environment is dynamic and that is what defines evolution. See the dynamic matrix chemical evolutionary hypothesis postulated by Chevrette, Barona Gómez, Hoskisson." **Response: Discussion/ Correction.** This point is well taken, and the paragraph has been modified to provide a more balanced discussion of possible natural function. The term 'primary function' has been removed, and the DMCE hypothesis briefly discussed in the new section on ecological drivers of diversity.

"e. Page 8, lines 6-9, similar to previous point: the only thing that could be concluded about the relationship between BGC diversity and biomolecular activity is limited to the bioassay used, and thus cannot be extrapolated into such strong evolutionary statement(s)."

Response: Clarification. This paragraph posits the idea that the topology of structure networks can provide information about the relative merit of different structural modifications in the environment, irrespective of knowledge about environmental function(s). Many evolutionary models for natural products suggest that selection is driven by function. The further away a molecule is from the optimal structure for those functions, the lower the pressure for retention. By definition therefore, molecules at the center of clusters are most likely to be those that are also close to the optimized structures for function, regardless of whether the true function(s) are known. The paragraph does not state this as fact but rather raises this idea as one worthy of further consideration and discussion.

"f. Page 10, lines 7 – 15. Indeed, the authors are aware of the biased in place. So it is hard for me to explain why these were not more carefully examined to avoid incongruent and misleading statements about the evolution of chemical diversity."

Response: Discussion/ Addition. As described above this topic has now been given its own section in the manuscript, and all sections of the paper reviewed and modified to provide a more balanced viewpoint. It is worth reiterating that this article was never intended to be a review of the evolutionary basis of natural products diversity, but rather is designed to examine what is currently known about structural diversity from a chemical perspective, and to use these data to explore questions surrounding past and future discovery trends in the field.

"3. Not sure where the MIC data of Figure 2 comes from and how these data was treated."

Response: Clarification. MIC data for figure 2 are extracted directly from the literature references reporting the discovery of each compound. Literature references have been added as a new column in the Cytoscape file for Figure 2 in the Data Availability link to Zenodo, and the figure caption for Figure 2 updated to indicate availability of Cytoscape file.

"4. The author miss altogether the important role of convergent evolution during synthesis of key functional moieties, for instance the beta lactam ring. Probably this is what they refer to in Figure 2? See opinion papers by Fischbach, Walsh, Ross and others on convergent evolution."

Response: Discussion. As discussed above, the objective of this outlook article is to discuss what proportion of theoretical chemical diversity has currently been isolated, not to suggest how these molecules evolved. This question, while valuable, is better suited to a different article written by domain specialists who can discuss the intricacies and considerable uncertainties in the topic with the depth and detail that the subject deserves.

"5. Within the final Outlook section, I found the second paragraph not informative and out of place (This manuscript is not about unnatural natural products, but quite the opposite)." **Response: Clarification.** Because ACS Central Science is a generalist journal it is expected that the readership will include researchers in related fields (e.g. synthetic organic and medicinal chemistry) as well as researchers in both academic and industrial settings. One conclusion from this work is that natural extracts are likely to provide access to only a small fraction of theoretically possible scaffolds for any compound class. This paper therefore provides clear, data-driven evidence that natural product and synthetic approaches to library development are complementary rather than

duplicative, and that medicinal chemistry (or biosynthetic engineering) can offer access to chemical space that is not functionally accessible from nature. This point is important because it could influence the design strategy or chemical composition of future (bio)synthetic library projects.

"6. Also in this section, ref 39 is wrong. While third paragraph lacks all together any reference to previous work or ideas being discussed."

Response: Discussion/ Correction. Reference 39 has been corrected and all references reviewed for accuracy. With respect to references for the third paragraph it is difficult to see many places where additional references could be sensibly added. The paragraph provides the author's personal opinion about the importance of developing open and accurate data repositories, and offers several suggestions of ways in which poor or incomplete data curation will hinder the field. The paragraph is not designed to provide a summary of existing resources, nor to review informatics platforms generated using these data. References to representative articles from other groups advocating for FAIR natural products data repositories have been added. More broadly, the number of references has increased from 49 in the original submission to 63 in the revised manuscript.

"7. Page 13, last paragraph includes companies that should not be mentioned as many are omitted. Same with NP research programs. Not a good approach for an outlook paper."

Response: Correction. References to example companies and programs have been removed as requested.

References

(1) Le, T. C.; Yang, I.; Yoon, Y. J.; Nam, S.-J.; Fenical, W. Ansalactams B–D Illustrate Further Biosynthetic Plasticity within the Ansamycin Pathway. *Org. Lett.* **2016**, *18* (9), 2256–2259.

(2) Sester, A.; Stüer-Patowsky, K.; Hiller, W.; Kloss, F.; Lütz, S.; Nett, M. Biosynthetic Plasticity Enables Production of Fluorinated Aurachins. *Chembiochem* **2020**, *21* (16), 2268–2273.

(3) Trendafilova, A.; Todorova, M.; Wolfram, E.; Peter, S.; Ivanova, V.; Danova, K. Unfolding Phenolics Biosynthetic Plasticity of Artemisia Alba Turra through Plant Tissue Culture Techniques. *Plant Cell Tissue Organ Cult.* **2024**, *157* (2). https://doi.org/10.1007/s11240024-02756-y.

(4) Aryal, N.; Chen, J.; Bhattarai, K.; Hennrich, O.; Handayani, I.; Kramer, M.; Straetener, J.; Wommer, T.; Berscheid, A.; Peter, S.; Reiling, N.; Brötz-Oesterhelt, H.; Geibel, C.; Lämmerhofer, M.; Mast, Y.; Gross, H. High Plasticity of the Amicetin Biosynthetic Pathway in Streptomyces Sp. SHP 22-7 Led to the Discovery of Streptcytosine P and Cytosaminomycins F and G and Facilitated the Production of 12F-Plicacetin. *J. Nat. Prod.* **2022**, *85* (3), 530–539.

(5) Gu, W.; Dong, S.-H.; Sarkar, S.; Nair, S. K.; Schmidt, E. W. The Biochemistry and Structural Biology of Cyanobactin Pathways: Enabling Combinatorial Biosynthesis. *Methods Enzymol.* **2018**, *604*, 113–163.
